# AKR1B1 and AKR1B10 as Potential Prognostic Biomarkers of Endometrial Cancer

**DOI:** 10.3390/ijms27177639

**Published:** 2026-08-26

**Authors:** Maja Novak Pušić, Špela Smrkolj, Tea Lanišnik Rižner

**Affiliations:** 1 Institute of Biochemistry and Molecular Genetics, Faculty of Medicine, University of Ljubljana, 1000 Ljubljana, Slovenia; maja.pusic@mf.uni-lj.si; 2 Department of Gynaecology and Obstetrics, Faculty of Medicine, University of Ljubljana, 1000 Ljubljana, Slovenia; spela.smrkolj@mf.uni-lj.si; 3 Division of Gynaecology and Obstetrics, University Medical Centre, 1000 Ljubljana, Slovenia

**Keywords:** endometrial carcinoma, plasma biomarkers, aldo-keto reductase, lymphovascular invasion, overall survival, recurrence-free survival

## Abstract

Endometrial cancer (EC) is the most common gynecological malignancy with rising incidence, yet reliable non-invasive prognostic biomarkers for preoperative risk stratification and treatment guidance are needed. This pilot study investigated whether plasma levels of aldo-keto reductase family 1 member B1 (AKR1B1) and aldo-keto reductase family 1 member B10 (AKR1B10) could serve as non-invasive prognostic biomarkers in EC. Plasma samples were collected preoperatively from 78 postmenopausal women with histologically confirmed EC alongside clinicopathological data including histological grade, depth of myometrial invasion, lymphovascular invasion, and metastatic status. AKR1B1 and AKR1B10 protein levels were quantified using enzyme-linked immunosorbent assays (ELISAs) in 72 and 64 plasma samples, respectively, and data were analyzed using R v4.3.0. Plasma AKR1B10 levels were significantly higher in Grade 3 compared to Grade 1–2 EC patients; and should be considered with caution given the limited Grade 3 sample size. A model combining both AKR1B1 and AKR1B10 with clinical variables (BMI, age, smoking, parity, hormone replacement therapy, previous use of contraceptives) showed limited discriminative ability for preoperative lymphovascular invasion prediction and is not suitable for clinical use in its current form. Survival analysis revealed that patients with plasma levels of both AKR1B1 and AKR1B10 below the median showed significantly better overall (*p* = 0.04, hazard ratio (HR) = 0.33, 95% confidence interval (CI): 0.10–1.01) and recurrence-free survival (*p* = 0.005, HR = 0.09, 95% CI: 0.02–0.49) compared to the remainder of the cohort. These preliminary, hypothesis-generating findings suggest the potential of circulating AKR1B proteins as candidate prognostic biomarkers in EC, requiring prospective validation in larger cohorts.

## 1. Introduction

Endometrial cancer (EC) is the most prevalent gynecological malignancy in industrialized countries [1,2]. Its incidence has been steadily increasing since the 1960s, primarily due to rising obesity rates and prolonged life expectancy [3]. In 2020 alone, 417,367 new cases and 97,370 related deaths were reported worldwide; in Europe, there were 130,051 new cases and 29,963 deaths [4]. Currently, no effective screening methods exist for EC, and diagnosis relies on minimally invasive or invasive surgical procedures accompanied by histopathological examination [5]. The discovery of specific biomarker(s) for EC could greatly facilitate non-invasive early detection and potentially improve clinical outcomes by enabling timely intervention before disease progression [6]. Moreover, reliable prognostic biomarkers for pre-operative patient stratification would support informing surgical decision-making and the tailoring of individualized therapeutic approaches [7]. Such prognostic biomarkers would ideally help predict disease recurrence, which occurs in approximately 15–20% of patients initially diagnosed without evidence of advanced disease.

In 2023, the International Federation of Gynecology and Obstetrics (FIGO) introduced a revised staging system for EC, incorporating molecular classifications to improve prognostic accuracy and guide treatment strategies. This integration has modified prognostic assessment by stratifying patients into different risk groups, allowing for a more individualized, patient-oriented treatment approach. The FIGO 2023 staging acknowledges the molecular heterogeneity of EC, including subtypes such as POLE ultramutated (POLEmut), mismatch repair deficient (MMRd), no specific molecular profile (NSMP), and p53 abnormal (p53abn), each carrying distinct prognostic implications. By providing more nuanced risk stratification, it offers significant potential to refine patient management, personalize therapeutic strategies, and ultimately improve outcomes for patients with EC [8]. In addition to molecular markers, the 2023 FIGO update also includes lymphovascular invasion (LVI) as a prognostic factor [8]. LVI is defined as the presence of tumor cells within endothelial-lined spaces outside the primary tumor mass [9] and has been associated with tumor recurrence [10,11], overall survival [12,13,14], and lymph node metastasis [15]. Importantly, LVI status can only be determined after hysterectomy and relies on histological analysis, which is subject to inter-observer variability [9]. Therefore, a non-invasive biomarker capable of predicting LVI preoperatively would enable earlier risk assessment in EC. Despite advancements in molecular and pathological classification, there remains a need for additional non-invasive biomarkers to further refine prognosis, optimize risk stratification, and enhance individualized therapeutic management in EC. Non-invasive biomarkers, such as circulating proteins measured in blood, offer several advantages over tissue-based biomarkers currently used in clinical practice. Unlike tissue biopsy, which is invasive and painful, blood sampling is minimally invasive, well tolerated, and easily repeatable, enabling longitudinal monitoring of disease progression and treatment response [16]. Furthermore, protein quantification by enzyme-linked immunosorbent assay (ELISA) represents a cost-effective and widely accessible approach suitable for routine clinical implementation [17].

It is known that enzymes of the aldo-keto reductase (AKR) subfamily 1B (AKR1B1 and AKR1B10) play an important role in the progression and chemoresistance of various types of cancer [18]. These monomeric enzymes catalyze the nicotinamide adenine dinucleotide phosphate (NADPH)-dependent reduction of carbonyl to hydroxyl groups in a variety of substrates [18,19]. AKR1B1 and AKR1B10 act on both endogenous and exogenous compounds and catalyze a range of reactions [20]. AKR1B1, also known as aldose reductase, acts on aldehydes produced by lipid peroxidation, prostaglandins, isoprenyl aldehydes, trans-retinal and various exogenous compounds [18,20,21]. AKR1B10 acts on trans-retinal and 9-cis-retinal, isoprenyl aldehydes, aldehydes derived from lipid peroxidation and a number of drugs [18,22,23]. Additionally, AKR1B10 has three known moonlighting functions: promotion of lipid synthesis, lysosomal-mediated excretion and suppression of autophagy [22]. AKR1B1 and AKR1B10 share several partially overlapping physiological functions [22]. Both enzymes catalyze the formation of retinol, thereby reducing the synthesis of proliferation-inhibiting retinoic acid; they reduce the isoprenyl aldehydes farnesal and geranylgeranial to the corresponding alcohols, which can be phosphorylated and used for the prenylation of small G-proteins, leading to proliferation; and they catalyze the degradation of toxic aldehydes derived from lipid peroxidation, thus reducing mutagenesis and carcinogenesis [18,20]. In the context of endometrial cancer, these functions may be particularly relevant because AKR1B1 has high catalytic efficiency for prostaglandin F2α (PGF2α) synthesis [24], while increased local formation of PGF2α has been reported in cancerous endometrium [20,25]. As PGF2α promotes proliferation, migration, and angiogenesis in endometrial cancer cells, AKR1B1-mediated prostaglandin metabolism may contribute to endometrial cancer progression [20]. In addition, AKR1B10 drives endometrial cancer growth by depleting retinoic acid (RA), effectively depriving the cell of the ligand required to activate retinoic acid receptor α(RARα) signaling. This metabolic depletion mimics the phenotypic effects observed in α(RARα) knockdown models, where the loss of receptor signaling blocks the anti-proliferative activities of RA and RA agonists like AM580 in endometrial cancer cells [26].

In normal physiology, AKR1B1 is predominantly expressed in the adrenal gland but is also detectable in all other tissues, while AKR1B10 is mainly expressed in the gastrointestinal tract, although it is present in many other tissues as well [27] (AKR1B1 and AKR1B10, available at https://www.proteinatlas.org/, accessed on 25 November 2025). Notably, expression of the *AKR1B1* and *AKR1B10* genes has also been detected in various cancer tissues [28], as shown by TCGA RNA sequencing data [27] (AKR1B1 and AKR1B10 available at https://www.proteinatlas.org/, accessed on 25 Novemeber 2025). Although *AKR1B1* and *AKR1B10* are normally overexpressed in cancer, they are downregulated in some cancer types [29].

We previously found lower mRNA levels of *AKR1B1* and higher mRNA levels of *AKR1B10* in cancer tissue compared to adjacent control tissue from 47 EC patients. In contrast, Western blot analysis showed lower protein levels of both AKR1B1 and AKR1B10 in cancer tissue [30]. Further immunohistochemical (IHC) staining of paraffin-embedded tissue sections from 101 patients with endometrioid EC confirmed lower levels of AKR1B1 and AKR1B10 in cancerous tissue compared to adjacent control endometrium [31]. In addition, Kaplan–Meier and Cox regression analyses showed longer overall and disease-free survival for patients with both AKR1B1 and AKR1B10 levels above the median, with hazard ratios of 0.4 and 0.3, respectively [31]. However, tissue-based biomarker assessment requires surgical specimens obtained postoperatively, precludes preoperative risk stratification, and reflects only local tumor expression rather than systemic disease burden. These limitations motivated the present investigation of circulating plasma protein levels as a non-invasive alternative.

The observed prognostic potential of AKR1B1 and AKR1B10 in EC tissue prompted us to quantify these in plasma samples, offering a non-invasive approach to their assessment. The primary objective of this pilot study was to determine whether plasma levels of AKR1B1 and AKR1B10 could serve as non-invasive prognostic biomarkers for EC. The secondary objective was to assess whether plasma levels of these proteins, in combination with patients’ clinical characteristics, could predict LVI status in EC patients preoperatively.

## 2. Results

### 2.1. Clinical Characteristics of Patients

**Figure 1:**
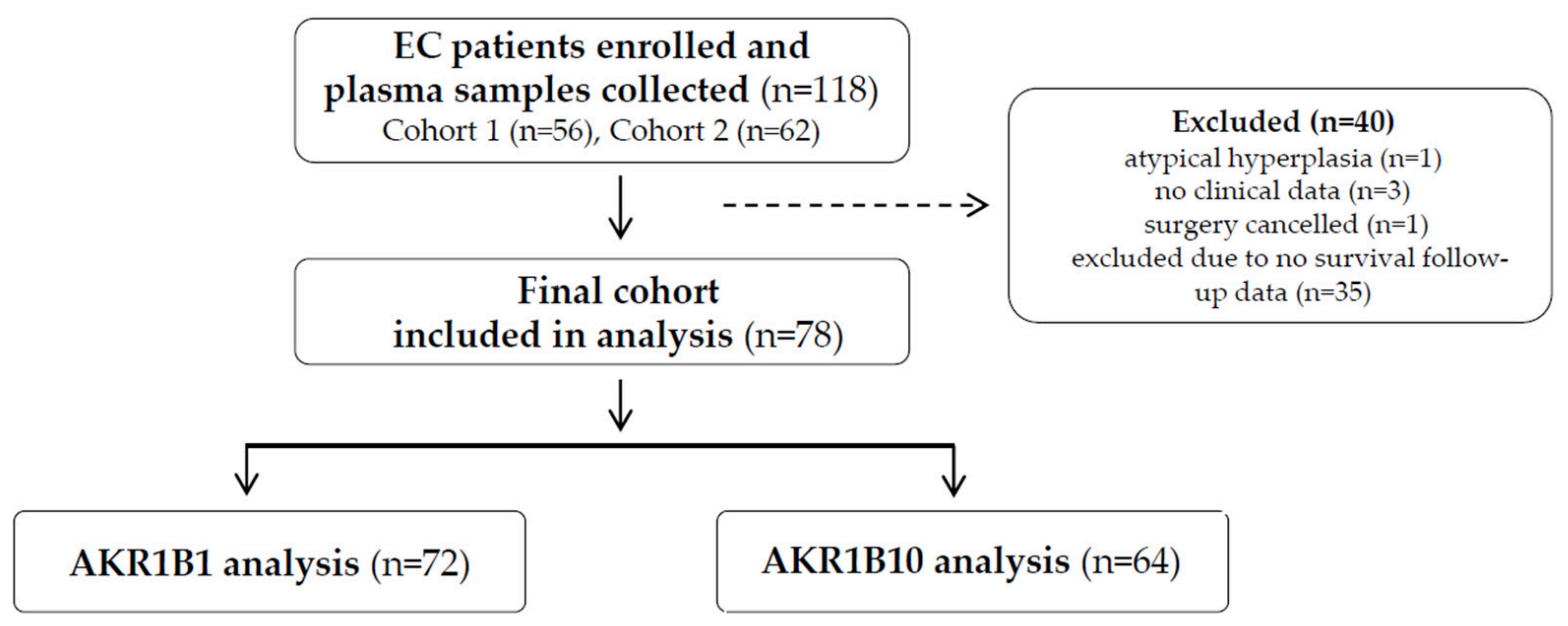
Flowchart of patient selection. Abbreviations: AKR1B1, aldo-keto reductase proteins family 1 member B1; AKR1B10, aldo-keto reductase family 1 member B10; EC, endometrial cancer.

In this study, plasma samples from 118 women with diagnosed EC were selected from two previously established cohorts. Five patients were excluded based on exclusion criteria: one patient had a final diagnosis of atypical hyperplasia, three were excluded due to lack of clinical data, and one had surgery canceled. Of the remaining 113 patients, 35 were further excluded due to the unavailability of survival follow-up data at the time of analysis, resulting in a final cohort of 78 patients (Figure 1). Among the 78 patients, 92% had endometrioid EC and 8% had serous cancer. Histological grading of the EC tissue samples revealed that 55% of patients were classified as Grade 1, 22% as Grade 2, and 15% as Grade 3. According to FIGO 2009, the patients were classified as follows: IA (55.1%), IB (23.1%), II (5.1%), IIIA (2.6%), IIIB (1.3%), IIIC (6.4%) and IVB (5.1%), with one patient lacking staging information. Regional lymph node metastasis and pelvic organ invasion were present in 10% of patients, while distant metastases were present in 5%. Myometrial invasion was absent in 16% of patients, 45% had <50% and 37% had >50% invasion into the myometrium. LVI was present in 27% of patients. The clinical characteristics of patients for each group are presented in Table 1.

A smaller number of patients were included in the measurement of AKR1B10 (*n* = 64) due to optimization of the ELISA method for detection of this protein. However, no statistically significant differences were observed in the clinical characteristics between the patients in the AKR1B10 and AKR1B1 groups (Table 1, *p*-values).

### 2.2. In Patients with Lymphovascular Invasion, AKR1B10 Levels Correlate with Age

**Figure 2:**
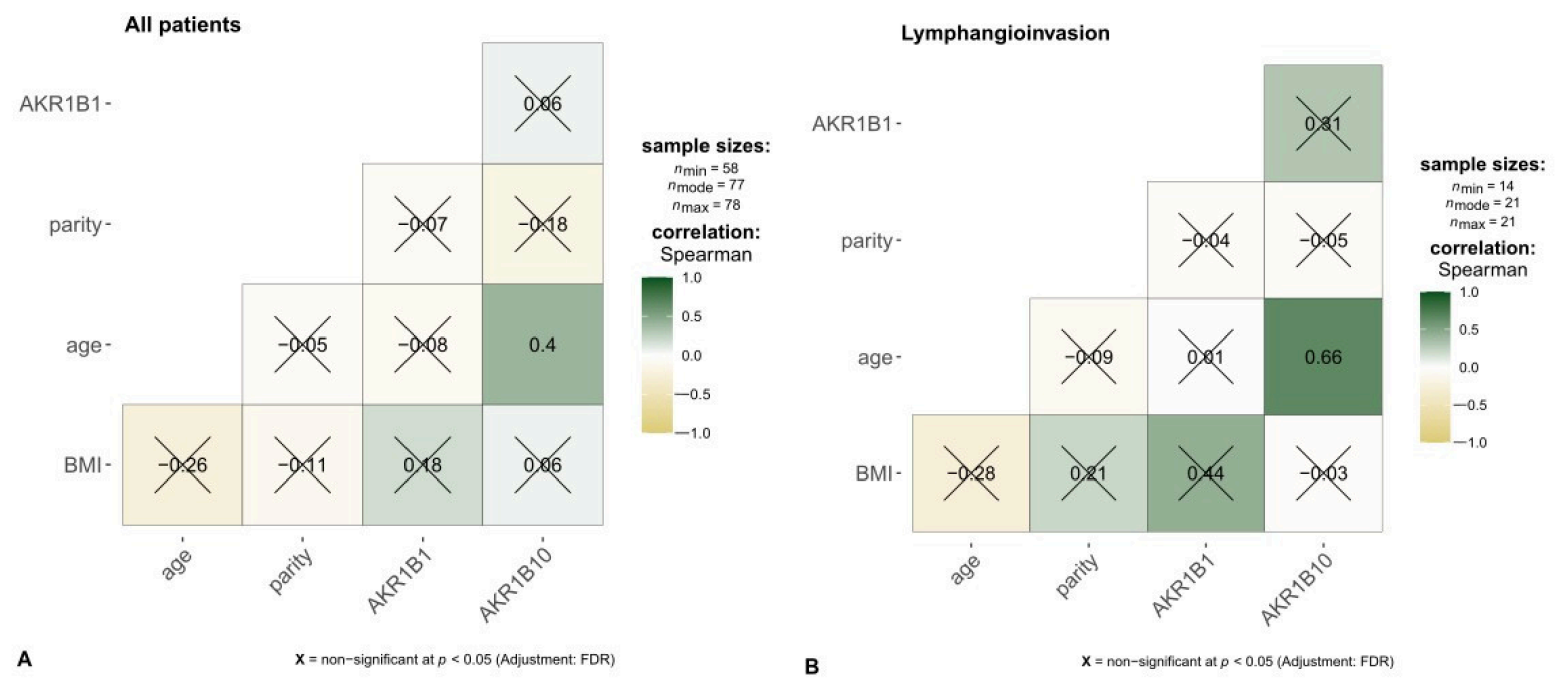
Spearman’s correlation between numerical predictors. Heatmap showing Spearman’s correlation coefficients among continuous clinical data and AKR1B1 and AKR1B10 plasma levels for all patients (**A**) and patients with lympovascular invasion (**B**). Darker colors indicate stronger correlations. *n* (all patients) = 78, *n* (lymphovascular invasion) = 21. Abbreviations: BMI, body mass index; AKR1B1, aldo-keto reductase proteins family 1 member B1; AKR1B10, aldo-keto reductase proteins family 1 member B10; *n*, number of patients.

Correlation testing between AKR1B1 and AKR1B10 protein levels and numerical variables (body mass index - BMI, age and parity) revealed a statistically significant correlation between AKR1B10 and age (Figure 2A, Spearman’s coefficient = 0.4, adjusted *p*-value < 0.05). When patients were grouped by LVI status, a significant correlation between AKR1B10 and age was found only in the LVI-positive group (Figure 2B, Spearman’s coefficient = 0.66, adjusted *p*-value = 0.0011).

### 2.3. Patients with Higher-Grade EC Have Significantly Higher AKR1B10 Levels

**Figure 3:**
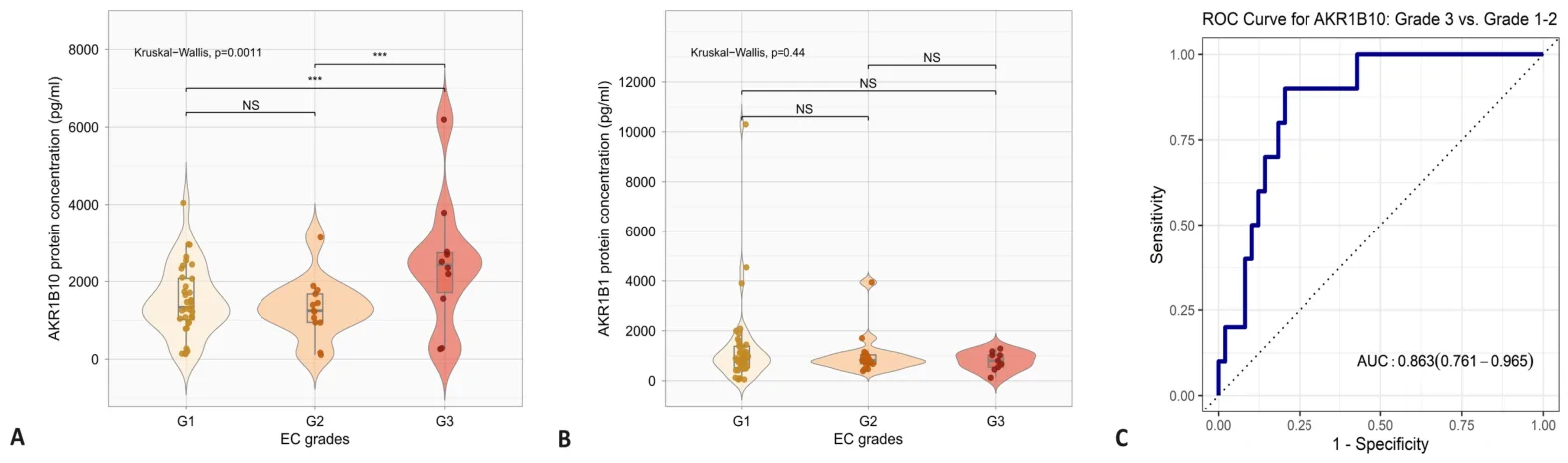
Plasma levels of AKR1B1 and AKR1B10 in endometrial cancer patients and ROC analysis of AKR1B10. (**A**,**B**) Distribution of AKR1B10 and AKR1B1 levels measured in plasma samples of patients with different grades of endometrial cancer; (**C**) ROC curve analysis of AKR1B10 distinguishing Grade 3 from Grades 1–2 EC patients. Statistical significance was assessed using Kruskal–Wallis test (*** *p* < 0.005). *n* (Grade 1) = 39, *n* (Grade 2) = 17, *n* (Grade 3) = 10. Abbreviations: EC, endometrial cancer; ROC, receiver operating characteristics; AKR1B1, aldo-keto reductase proteins family 1 member B1; AKR1B10, aldo-keto reductase proteins family 1 member B10; AUC, Area Under the Curve; G1, Grade 1; G2, Grade 2; G3, Grade 3; NS, non-significant; *n*, number of patients.

AKR1B10 levels were significantly higher in plasma from Grade 3 EC patients compared to Grades 1 and 2 (Figure 3A), while this trend was not observed for AKR1B1 (Figure 3B). Receiver operating characteristics (ROC)curve analysis showed an Area Under the Curve (AUC) of 0.86 (95% confidence interval (CI): 0.76–0.97, cut-off: 2146.95 pg/mL, sensitivity: 90.0%, specificity: 79.5%) for AKR1B10 in distinguishing Grade 3 from Grade 1–2 patients (Figure 3C). Additionally, logistic regression with 5-fold cross-validation was performed on the complete dataset without prior imputation of missing values, using either each protein alone or both proteins together. This analysis showed that only AKR1B10 alone was a significant predictor (*p* = 0.0038) of EC grade when comparing Grade 1 and 2 patients versus Grade 3 patients (Supplementary Table S2). However, these findings should be interpreted as preliminary and hypothesis-generating given the very small Grade 3 subgroup (*n* = 10). Furthermore, the AUC estimate may be inflated due to overfitting, as reflected by the relatively wide confidence interval (95% CI: 0.76–0.97).

### 2.4. A Predictive Model Combining Plasma AKR1B1 and AKR1B10 with Clinical Variables Shows Limited Performance for Lymphovascular Invasion Prediction

To test whether AKR1B10 and AKR1B1 have prognostic potential, their plasma levels were analyzed in EC patients stratified by type, FIGO stage, presence of LVI, depth of myometrial invasion, and presence of metastases. Data analysis revealed no statistically significant differences in plasma levels of AKR1B10 and AKR1B1 between the studied groups (Supplementary Figures S1 and S2).

**Figure 4:**
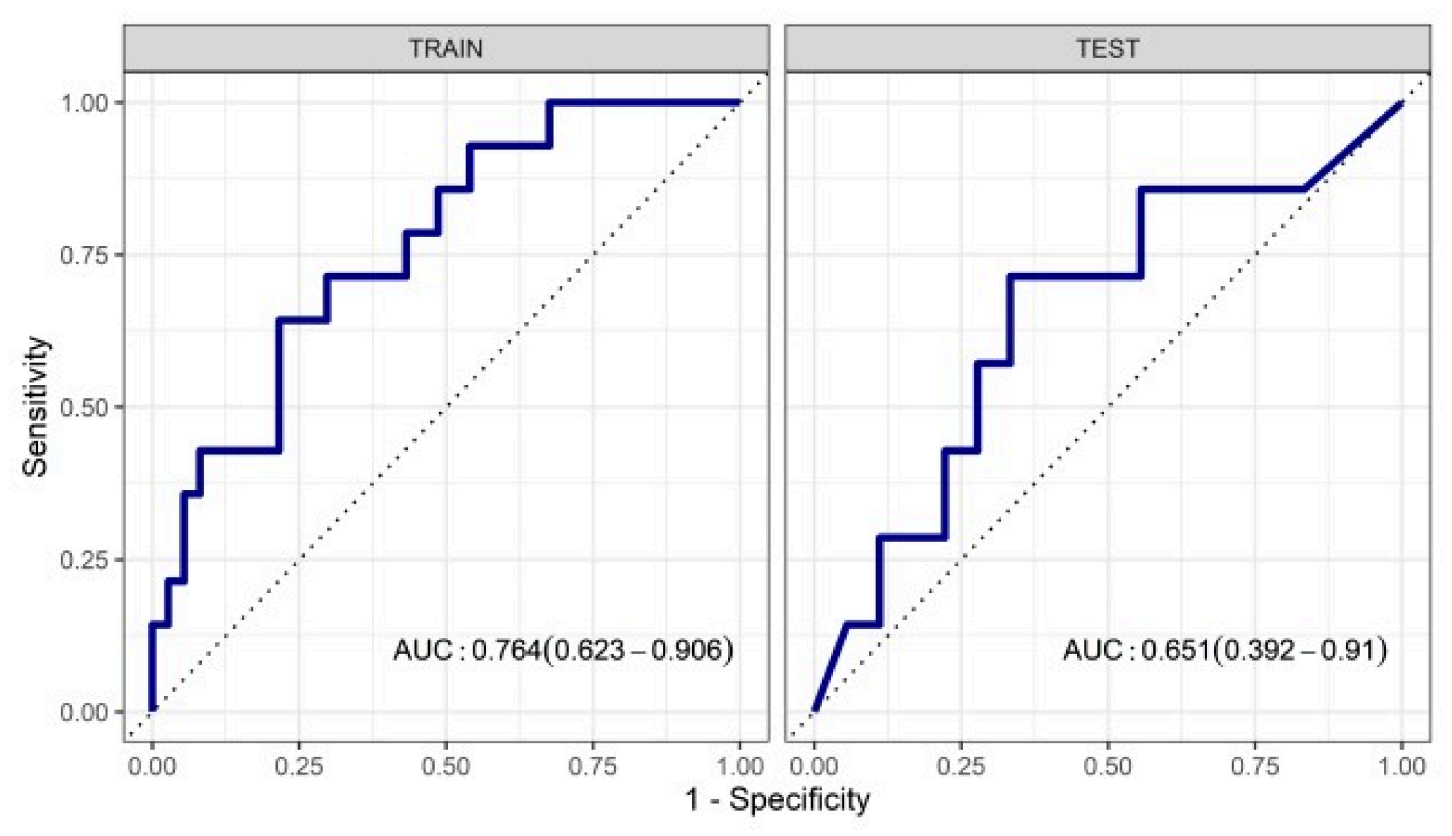
Receiver operating characteristic (ROC) curves and Area Under the Curve (AUC) for predicting lymphovascular invasion using logistic regression. ROC curves and corresponding AUC for the training set performed using “SMOTE” method and test set. Models were generated using logistic regression to predict lymphovascular invasion status of patients. *n* (training) = 52, *n* (test) = 26. Abbreviations: AUC, Area Under the Curve; SMOTE, Synthetic Minority Over-Sampling Technique; *n*, number of patients.

Since LVI is associated with poorer survival in EC patients, we tested whether AKR1B1 and AKR1B10, together with other clinical characteristics of patients, could be used to predict LVI. To model LVI, the dataset was split into a training set (66%) and a test set (33%), and the following variables were used: BMI, age, smoking, parity, hormone replacement therapy, previous use of contraceptives and plasma levels of AKR1B1 and AKR1B10. Model performance on the training set across different sampling methods was evaluated using the AUC of the ROC curves (Supplementary Figure S3). The Synthetic Minority Over-Sampling Technique (SMOTE) method yielded the best results in the training set, with an AUC of 0.764 (Figure 4). In the test set, this model achieved an AUC of 0.651, a sensitivity of 42.9%, a specificity of 77.8%, and an accuracy of 68%, with a precision, recall, and F1 score of 0.429. Logistic regression coefficients are shown in Supplementary Table S3.

### 2.5. Combined Reduced Plasma Levels of AKR1B1 and AKR1B10 Associate with Better Overall and Recurrence-Free Survival

**Figure 5:**
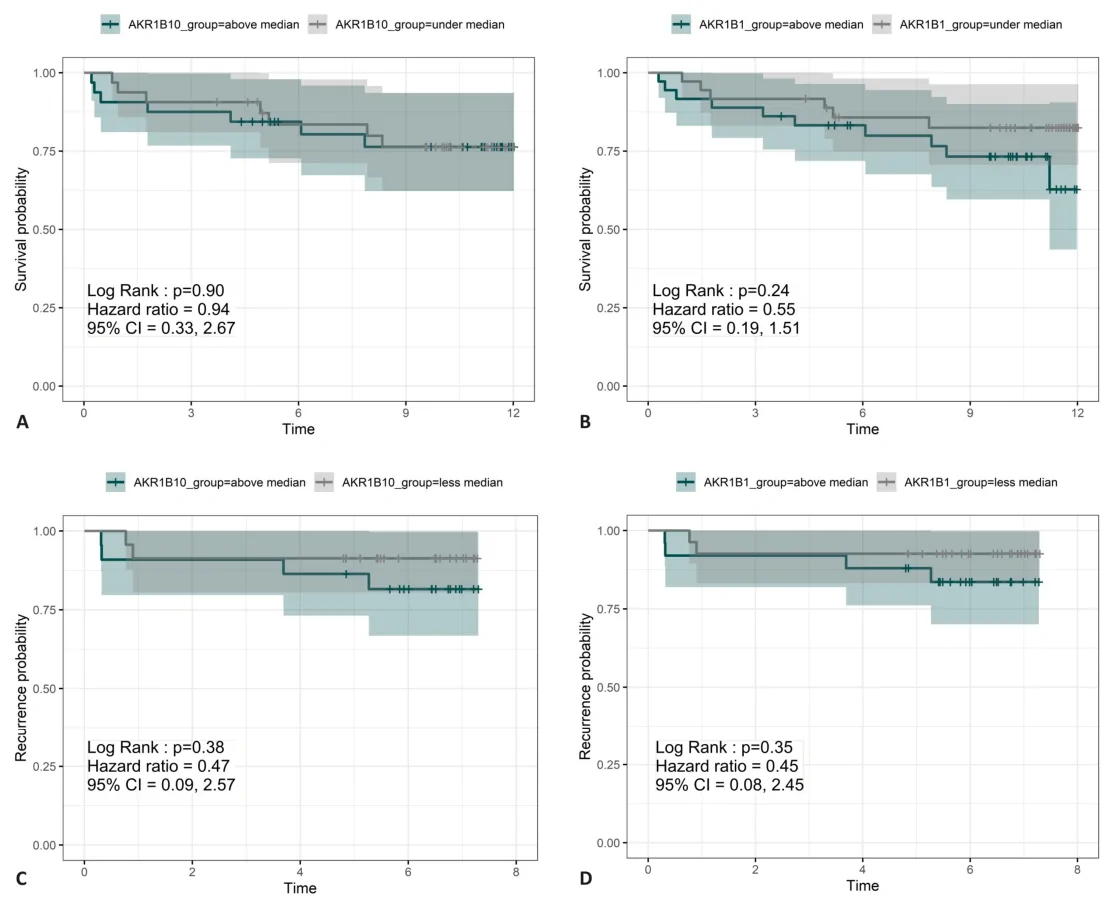
Kaplan–Meier overall survival and recurrence-free survival curves based on plasma levels of AKR1B10 and AKR1B1. (**A**,**B**) Overall survival and (**C**,**D**) recurrence-free survival stratified by median plasma levels of AKR1B10 (**A**,**C**) and AKR1B1 (**B**,**D**) in endometrial cancer patients. Patients are divided into two groups: those with plasma levels of AKR1B1 or AKR1B10 above the median (*n* (AKR1B1) = 35, *n* (AKR1B10) = 32), and those with protein levels below the median (*n* (AKR1B1) = 36, *n* (AKR1B10) = 32). The x-axis indicates the time in years elapsed since initial diagnosis, while the y-axis indicates the survival and recurrence probability. Statistical significance was assessed using the log-rank test. Hazard ratios (HRs) and 95% confidence intervals (CIs) are indicated for each analysis. *p*-value < 0.05 was considered significant. Abbreviations: AKR1B1, aldo-keto reductase proteins family 1 member B1; AKR1B10, aldo-keto reductase proteins family 1 member B10; *n*, number of patients.

To investigate the association between plasma levels of AKR1B10 and AKR1B1 with survival and recurrence, EC patients were divided based on the median expression levels of AKR1B1 and AKR1B10. Kaplan–Meier analysis showed no significant differences in overall survival (*p* = 0.9 for AKR1B10, *p* = 0.24 for AKR1B1) or recurrence (*p* = 0.38 for AKR1B10, *p* = 0.35 for AKR1B1) between groups (Figure 5A–D). In multivariable Cox proportional hazards models including smoking, parity, use of hormonal therapy, use of oral contraception in the past, and LVI, only LVI emerged as a significant predictor of overall survival (Table 2), while AKR1B1 showed a trend toward shorter survival at lower plasma levels (*p* = 0.075) (Table 2 and Table 3). To explore potential confounding by LVI, additional sensitivity analyses were performed with models excluding this variable. When LVI was excluded, AKR1B1 plasma levels below the median showed an impact on overall survival (Table 3). These findings suggest that the prognostic effect of AKR1B1 may be partially mediated by, or correlated with, LVI status. Cox models for the recurrence analysis showed no impact of any of the covariates on the outcome, with or without LVI as a predictor (Supplementary Tables S4 and S5). Cox proportional hazards models for overall survival showed wide confidence intervals for several covariates, reflecting limited statistical precision (Table 2 and Table 3). Cox models for recurrence failed to produce stable estimates due to the very small number of recurrence events (*n* = 6), and the resulting hazard ratios and confidence intervals are unreliable (Supplementary Tables S4 and S5).

**Figure 6:**
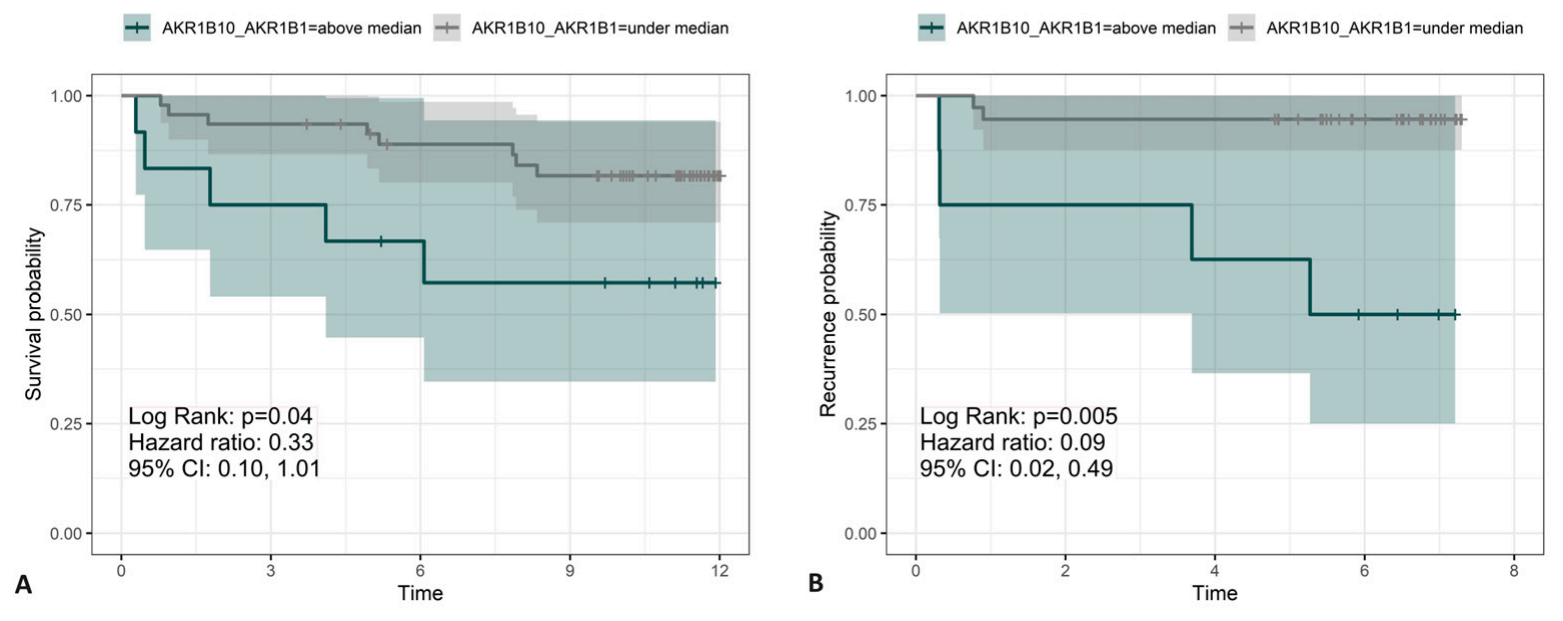
Kaplan–Meier overall survival and recurrence-free survival curves based on plasma levels of both AKR1B10 and AKR1B1. (**A**) Overall survival and (**B**) recurrence-free survival in endometrial cancer patients stratified by combined plasma levels of AKR1B1 and AKR1B10. Patients are divided into two groups: those with plasma levels of both AKR1B1 and AKR1B10 below the median (*n* = 46) and those above the median (*n* = 32). The x-axis indicates the time in years elapsed since initial diagnosis, while the y-axis indicates the survival and recurrence probability. Statistical significance was assessed using the log-rank test. Hazard ratios (HRs) and 95% confidence intervals (CIs) are indicated for each analysis. *p*-value < 0.05 was considered statistically significant. Abbreviations: AKR1B1, aldo-keto reductase proteins family 1 member B1; AKR1B10, aldo-keto reductase proteins family 1 member B10; *n*, number of patients.

However, Kaplan–Meier analysis of patients with both AKR1B10 and AKR1B1 plasma levels below the median showed significantly better overall survival (*p* = 0.041, HR = 0.33, 95% CI: 0.10–1.01) and recurrence-free survival (*p* = 0.005, HR = 0.09, 95% CI: 0.02–0.49) compared to the rest of the cohort (Figure 6A,B).

## 3. Discussion

This pilot study is the first to investigate plasma levels and the prognostic potential of AKR1B1 and AKR1B10 in patients with EC. We found that AKR1B10 levels correlate significantly with age in patients with LVI. While this association might help identify a subset of patients at increased risk for aggressive disease and poorer outcomes, the finding requires prospective validation before any clinical implications can be drawn.

We also found significantly increased AKR1B10 plasma levels in patients with endometrioid Grade 3 EC compared to Grades 1 and 2, with an AUC of 0.86. However, due to the small number of patients with high-grade EC, this result should be interpreted as a hypothesis-generating preliminary finding. Due to the small sample size of Grade 3 patients (*n* = 10), the AUC estimate is subject to overfitting, reflected by the relatively wide confidence interval (95% CI: 0.76–0.97). Validation in an independent cohort of at least 50–100 Grade 3 patients is required before any conclusions about clinical utility can be drawn. In exploratory survival and recurrence analyses patients with AKR1B1 and AKR1B10 levels below the median value showed statistically significantly better overall survival (*p* = 0.04, HR = 0.33, 95% CI: 0.10–1.01) and recurrence-free survival (*p* = 0.005, HR = 0.09, 95% CI: 0.02–0.49), suggesting that combined elevation of both proteins may have greater prognostic value than either protein alone.

Additionally, we explored the potential utility of combining plasma AKR1B1 and AKR1B10 levels with clinical variables to predict LVI non-invasively. The best-performing model showed moderate discriminative ability in the training set (AUC = 0.764), which diminished in the test set (AUC = 0.651, sensitivity = 42.9%, specificity = 77.8%), and is therefore unsuitable for preoperative LVI prediction without further optimization. Several factors may explain this limited performance: the relatively small sample size with only 21 LVI-positive patients, class imbalance between LVI-positive and LVI-negative groups despite SMOTE Over-Sampling, and the restricted number of candidate proteins included in the model. Expanding the feature set with additional plasma proteins or clinical variables may be needed to develop a clinically useful predictive model for LVI.

We previously found significantly lower immunohistochemical levels of AKR1B1 and AKR1B10 in EC tissue compared to adjacent control endometrium, with a trend toward better survival in patients with higher IHC levels [31]. Although no statistically significant differences in survival were observed when AKR1B1 or AKR1B10 were examined individually, Cox analysis identified combined AKR1B1 and AKR1B10 staining above the median as a statistically significant predictor of better overall survival [31]. The current plasma findings contrast with these tissue results. In plasma, combined levels of both proteins below the median were associated with better overall and recurrence-free survival. Such tissue–plasma discordance has also been described for other cancer-associated proteins, including E-cadherin, where increased circulating soluble E-cadherin may occur alongside reduced tissue expression, reflecting increased protein release into the circulation [32]. This discordance is not unexpected, as circulating protein levels reflect contributions from multiple tissues, including tumor, adjacent normal endometrium, and other systemic sources, and are further influenced by differential protein shedding, tissue injury or tumor necrosis, plasma stability, and protein turnover [33,34]. Systemic conditions may provide an additional source of variation, as AKR1B1 is involved in metabolic pathways associated with obesity and diabetes, while AKR1B10 has also been associated with metabolic and inflammatory conditions [35,36]. Thus, higher plasma AKR1B1 and AKR1B10 levels may reflect a combination of tumor-related and systemic processes rather than higher tumor expression alone. Future studies simultaneously measuring tissue expression and plasma protein levels in the same patients are needed to clarify this relationship.

Most studies that investigated AKR1B1 and AKR1B10 as diagnostic or prognostic biomarkers were carried out on tissue samples. AKR1B1 and AKR1B10 are frequently overexpressed in cancer tissues, including breast cancer, where IHC levels are found to be elevated compared to benign tissue [37]. High AKR1B10 expression has been linked to poorer survival in gastric cancer, while in colorectal cancer [38], high AKR1B1 and low AKR1B10 expression are associated with worse prognosis [39]. Published studies suggest opposing roles for these proteins, with only AKR1B1 promoting cell proliferation, cell cycle progression, cell motility, and NFκB activation [40]. The unknown role of AKR1B1 and AKR1B10 in endometrial cancer calls for further study but does not exclude their potential as prognostic biomarkers.

To date, AKR1B10 has been measured in blood samples from patients with breast [41] and hepatocellular carcinoma [42] and patients with non-alcoholic steatohepatitis [43]. In addition, recently AKR1B1 and AKR1B10 were detected in the blood of patients with various types of cancer, including ovarian, breast, and cervical cancer, using proximity extension assay with the Olink Explore and SomaScan platforms. However, neither protein has previously been measured in the blood of EC patients (AKR1B1 and AKR1B10 available at https://www.proteinatlas.org/, accessed on 25 November 2025). Although AKR1B10 has shown strong diagnostic characteristics in previous studies on breast and liver cancer, its expression across multiple malignancies suggests limited disease specificity. Although AKR1B10 is overexpressed in various cancers, it has also been reported to be downregulated in colorectal cancer, indicating that its expression may vary substantially across tumor types and may reflect broader tumor-associated metabolic changes rather than a cancer-specific process. This is particularly relevant in the context of endometrial cancer, given the shared molecular features between endometrial and colorectal cancers, including the importance of mismatch repair deficiency and Lynch syndrome. Thus, AKR1B10 is unlikely to be suitable as a standalone diagnostic or screening biomarker for endometrial cancer. However, disease specificity may be less critical in this context, as the intended application of plasma AKR1B10 is prognostic rather than diagnostic, and this is to non-invasively predict disease course and inform selection or adjustment of the surgical procedure.

In this context, our study evaluated AKR1B10 and AKR1B1 as potential prognostic biomarkers of EC, focusing on their association with clinical outcomes (such as LVI), to contribute to more accurate risk stratification and personalized treatment planning. Further validation in larger, independent cohorts is needed to establish the specificity and clinical utility of these prognostic associations, either alone or in combination with other biomarkers.

The strengths of this study include standardized preoperative blood collection using strict standard operating procedure (SOP), detailed clinical and lifestyle data collection, and thorough data analysis combining blood levels with clinicopathological and lifestyle data. The weaknesses of the study include the small sample size of the high-grade EC patient group, the lack of samples from different geographical regions (no validation in an external cohort), and the potential for selection bias due to the limited patient cohort. Additional limitations include the single-center design, which limits generalizability, the small number of outcome events, which reduces the precision of Cox regression estimates, and the lack of a formal sample size calculation. As a single-center study conducted in a Slovenian population, the generalizability of these findings to other ethnicities and healthcare settings requires evaluation in future multicenter studies.

In summary, this pilot study provides preliminary evidence that plasma AKR1B10 may differentiate higher-grade from lower-grade EC, and thatcombined plasma levels of both AKR1B1 and AKR1B10 below median may be associated with better survival outcomes. These hypothesis-generating findings require validation in larger multicenter cohorts, incorporating additional clinical variables to refine prognostic models. Moreover, genes (Supplementary Table S6) or proteins correlated with AKR1B1 and AKR1B10 could be included as part of multimarker panels for the prognosis of LVI and EC. Additionally, future prospective studies could investigate whether these circulating proteins hold any additional prognostic value when analyzed in combination with the updated 2023 FIGO molecular staging system.

## 4. Materials and Methods

### 4.1. Study Population and Sample Collection

This study was conducted and reported in accordance with the REporting recommendations for tumour MARKer prognostic studies (REMARK) guidelines for reporting of tumor marker prognostic studies (Supplementary Table S1). Patients enrolled at the Department of Obstetrics and Gynecology, University Medical Centre Ljubljana, Slovenia, between June 2012 and October 2020 were included in this study retrospectively. The study was approved by the National Medical Ethics Committee of the Republic of Slovenia (Nos. 0120-515/2017/4 and 0120-541/2019/7). Inclusion criteria were postmenopausal women with histologically confirmed EC who underwent total hysterectomy. Menopause was typically diagnosed clinically after a woman experienced 12 consecutive months without a menstrual period, indicating the end of reproductive capacity. Exclusion criteria were the presence of other malignancies, withdrawal of consent, or cancelation of surgery for any other reason. All patients signed a written informed consent form before participating in the study, and a dedicated gynecologist recorded the relevant clinical and gynecological data. The patient’s preoperative blood samples were collected following a strict SOP. Briefly, 6 mL of blood was collected by venipuncture using BD Vacutainer K2 EDTA tubes (Cat. No.: 367864, BD Medical, Franklin Lakes, NJ, USA). The collected blood samples were centrifuged at 1400× *g* for 10 min at 4 °C within 1 h after collection. The plasma was collected, mixed several times, transferred to 200 μL aliquots and stored at −80 °C for further analysis.

### 4.2. Enzyme-Linked Immunosorbent Assay (ELISA)

The plasma samples (undiluted) from 72 patients were analyzed using the Human AKR1B1 ELISA kit (Cat. No#: NBP2-60563, Lot#: 093042204, Novus Biologicals, Centennial, CO, USA), while plasma samples from 64 patients were diluted 1:5 and AKR1B10 was measured using the AKR1B10 ELISA kit (Cat. No#: NBP2-69860, Lot#: KL03N80X9364, Novus Biologicals, Centennial, CO, USA), following the manufacturer’s instructions. All samples were thawed only once. The manufacturer reported mean intra-assay coefficient of variation (CV) for AKR1B1 of 3.7% (*n* = 3 samples, 20 replicates) and mean inter-assay CV of 8.7% (*n* = 3 samples, 20 assays). The mean intra-assay CV for AKR1B10 was 5.3% (*n* = 3 samples, 20 replicates) and the mean inter-assay CV was 4.8% (*n* = 3 samples, 20 assays). In our study, inter-assay variability, assessed over two independent runs, showed CVs of 6.46% and 6.35% for AKR1B10, and 1.77% and 9.58% for AKR1B1, confirming good reproducibility of both assays. To minimize analytical bias, ELISAs were performed blinded to patients’ clinicopathological characteristics.

### 4.3. Statistical Analysis

No formal sample size calculation was performed, as no prior data on plasma AKR1B1 or AKR1B10 levels in EC patients were available to inform power estimation. The sample size was determined by the availability of plasma samples collected from twopatient cohorts during routine clinical care. Statistical analysis of clinical and ELISA data was performed using GraphPad Prism 9.3 (GraphPad Software, San Diego, CA, USA). The Shapiro–Wilk test was used to assess data normality. For comparisons between two groups, the unpaired t-test or Mann–Whitney test was used; for three or more groups, one-way ANOVA or Kruskal–Wallis test with Dunn’s multiple comparisons was applied. Fisher’s exact test, Chi-squared test, or Chi-squared test for trend was used for categorical variables. Statistical significance was set at *p* < 0.05.

Spearman’s correlations, ROC curve analysis, logistic regression model, survival analyses, and Cox proportional hazards models were performed using R version 4.3.0 [44]. Spearman’s correlations were assessed for numerical clinical characteristics in the entire dataset and based on LVI status. The Benjamini–Hochberg procedure controlled the false discovery rate for multiple comparisons, with significance set at *p* = 0.05.

The logistic regression model was trained on a dataset of 78 samples, split into 66% training and 33% test sets, stratified by cohort. Predictor variables were selected based on their known or potential association with LVI and EC prognosis as reported in the literature. Age and BMI were included as established clinical risk factors for EC, while smoking, parity, hormonal therapy, and previous use of oral contraceptives were included as lifestyle and reproductive variables with known and potential influence on EC biology. Plasma AKR1B1 and AKR1B10 levels were included as the primary biomarkers of interest. Missing values were handled using a single imputation approach [45]. For categorical variables, missing entries were replaced with the most frequent category from the training set and applied consistently to both training and test sets. For continuous variables, missing values were imputed using the median of the training set, which was likewise applied to both the training and test sets. Most continuous and categorical variables had less than 7% missing data. A 5-fold cross-validation approach was used to predict LVI status (seed = 1001). To address class imbalance, various sampling strategies were evaluated, including downsampling, upsampling, ROSE (Random Over-Sampling Examples), SMOTE, and no resampling (“none”). Model performance on the training set was evaluated using ROC and AUC. The final model (sampling method SMOTE), selected based on the highest AUC, was tested on the test set with metrics including accuracy, sensitivity, specificity, positive and negative predictive values, and AUC. Model coefficients, standard deviations, 95% CIs, and *p*-values were also extracted.

Survival and recurrence analyses were performed using the Kaplan–Meier estimator to estimate survival curves for different groups based on plasma levels of AKR1B1, AKR1B10, and LVI. Patients were divided into two groups based on the median concentrations of AKR1B1 (919.03 pg/mL) and AKR1B10 (1664.21 pg/mL): those with concentrations above the median and those below the median. The median was selected as the cut-off value to ensure balanced group sizes in this exploratory analysis.

The log-rank test was used to compare survival distributions between groups. Additionally, Cox proportional hazards regression assessed the impact of continuous and categorical covariates (age, BMI, smoking, parity, hormonal therapy, LVI) on time-to-event outcomes. To explore potential confounding by LVI, additional sensitivity analyses were performed with models excluding this variable. HRs and 95% CIs were calculated. Survival analyses were performed without prior imputation of data. Vital status and recurrence were obtained from the national oncology registry on a fixed censoring date (1 July 2024). Patients alive without recurrence at this date were right-censored, and censoring was assumed to be non-informative. During a median follow-up of 10.1 years (IQR: 5.1–11.2), 17 deaths and 6 recurrences were recorded among the 78 enrolled patients.

## Figures and Tables

**Table 1:** Clinical characteristics of patients included in the study.

	Characteristic	AKR1B10 *N* = 64	AKR1B1 *N* = 72	*p*-Value
Age (years)	mean ± SD	65.7 ± 7.9	66.8 ± 9.5	0.93 ^1^
Body mass index (kg/m^2^)	mean ± SD	31.8 ± 7.1	32.2 ± 7.0	0.76 ^2^
Smoking status (*n* (%))	Nonsmoker	50 (78%)	57 (79%)	0.96 ^4^
	Smoker	5 (8%)	5 (7%)	
	Former smoker	8 (12.5%)	9 (13%)	
	NA	1 (1.5%)	1 (1%)	
Parity status (*n* (%))	Nulliparous	8 (12.5%)	9 (13%)	0.85 ^4^
	Primiparous	13 (20%)	12 (17%)	
	Multiparous	42 (66%)	50 (70%)	
	NA	1 (1.5%)	1 (1%)	
Hormonal replacement therapy (*n* (%))	Yes	5 (8%)	4 (5%)	0.73 ^3^
	No	43 (67%)	50 (70%)	
	NA	16 (25%)	18 (25%)	
Previous use of oral contraception (*n* (%))	Yes	12 (19%)	14 (19%)	>0.99 ^3^
	No	33 (51%)	36 (50%)	
	NA	19 (30%)	22 (31%)	
Endometrial cancer type (*n* (%))	Type I	43 (67%)	48 (67%)	>0.99 ^3^
	Type II	21 (33%)	24 (33%)	
Endometrial cancer histology (*n* (%))	Endometrioid	59 (92%)	66 (92%)	>0.99 ^3^
	Serous	5 (8%)	6 (8%)	
	Mixed	/	/	
	Other	/	/	
Histological grade (*n* (%))	Well differentiated G1	36 (56%)	39 (54%)	0.88 ^4^
	Moderately differentiated G2	13 (20%)	17 (24%)	
	Poorly differentiated G3	10 (16%)	10 (14%)	
	NA	5 (8%)	6 (8%)	
FIGO stage (2009) (*n* (%))	IA	34 (53.1%)	42 (58%)	0.61 ^4^
	IB	16 (25%)	16 (22%)	
	II	3 (4.7%)	2 (3%)	
	IIIA	2 (3.1%)	2 (3%)	
	IIIB	1 (1.5%)	1 (1%)	
	IIIC	4 (6.3%)	5 (7%)	
	IVB	4 (6.3%)	3 (4%)	
	NA	/	1 (1%)	
Myometrial invasion (*n* (%))	No invasion	8 (13%)	13 (18%)	0.42 ^4^
	<50% myometrium	31 (48%)	37 (51%)	
	>50% myometrium	25 (39%)	21 (29%)	
	NA	/	1 (1%)	
Lymphovascular invasion (*n* (%))	Yes	21 (33%)	15 (21%)	0.17 ^3^
	No	43 (67%)	55 (76%)	
	NA	/	2 (3%)	
Metastasis (*n* (%))	Distant metastasis	4 (6%)	4 (6%)	0.71 ^4^
	Regional lymph node metastasis	5 (8%)	5 (7%)	
	Pelvic organ invasion	3 (5%)	2 (3%)	
	none	52 (81%)	60 (83%)	
	NA	/	1 (1%)	

^1^ Unpaired *t*-test. ^2^ Mann–Whitney test. ^3^ Fisher’s exact test. ^4^ Chi-squared test or Chi-squared test for trend. Abbreviations: AKR1B1, aldo-keto reductase proteins family 1 member B1; AKR1B10, aldo-keto reductase family 1 member B10; EC, endometrial cancer; SD, standard deviation; NA, not available; FIGO, International Federation of Gynecology and Obstetrics; n, number of patients.

**Table 2:** Cox proportional hazards model for survival analysis with lymphovascular invasion status as predictor and protein concentrations as groups.

Variable	Hazard Ratio	Lower 95% CI	Upper 95% CI	*p*-Value
Age	1.322	0.980	1.785	0.068
BMI	1.120	0.856	1.464	0.408
Smoker—Past	0.491	0.012	20.032	0.707
Smoker—Yes	0.000	0.000	Inf	0.999
Hormonal therapy—Yes	6.106	0.047	788.252	0.466
AKR1B10_group under median	8.185	0.111	604.449	0.338
AKR1B1_group under median	0.061	0.003	1.329	0.075
Parity	1.022	0.171	6.119	0.981
Lymphovascular invasion—Yes	30.998	1.009	952.711	**0.049**

Abbreviations: BMI, body mass index; AKR1B1, aldo-keto reductase proteins family 1 member B1; AKR1B10, aldo-keto reductase proteins family 1 member B10; CI, confidence interval; Inf, Infinity. Bold values indicate statistically significant results (*p* < 0.05).

**Table 3:** Cox proportional hazards model for survival analysis without lymphovascular invasion status as predictor and protein concentrations as groups.

Variable	Hazard Ratio	Lower 95% CI	Upper 95% CI	*p*-Value
Age	1.252	0.994	1.578	0.056
BMI	1.027	0.815	1.294	0.822
Smoker—Past	0.155	0.002	9.983	0.381
Smoker—Yes	0.000	0.000	Inf	0.999
Hormonal therapy—Yes	43.763	0.188	10,191.622	0.174
AKR1B10_group under median	0.607	0.039	9.469	0.722
AKR1B1_group under median	0.044	0.002	0.920	**0.044**
Parity	1.957	0.345	11.085	0.448

Abbreviations: BMI, body mass index; AKR1B1, aldo-keto reductase proteins family 1 member B1; AKR1B10, aldo-keto reductase proteins family 1 member B10; CI, confidence interval. Inf, Infinity. Bold values indicate statistically significant results (*p* < 0.05).

## Data Availability

The data supporting the conclusions of this article will be made available by the authors upon request.

## Supplementary Materials

The following supporting information can be downloaded at: https://www.mdpi.com/article/10.3390/ijms27177639/s1.

## Abbreviations

The following abbreviations are used in this manuscript:

AKR1B	Aldo-keto reductase subfamily 1B
AKR1B1	Aldo-keto reductase proteins family 1 member B1
AKR1B10	Aldo-keto reductase proteins family 1 member B10
ANOVA	Analysis of Variance
AUC	Area Under the Curve
BMI	Body mass index
CAT.NO	Catalogue number
CI	Confidence intervals
CV	Coefficient of variation
EC	Endometrial cancer
ELISA	Enzyme-linked immunosorbent assay
FIGO	International Federation of Gynecology and Obstetrics
G1	Grade 1
G2	Grade 2
G3	Grade 3
HR	Hazard Ratio
IHC	Immunohistochemistry
Inf	Infinity
K2 EDTA	Dipotassium ethylene diamine tetraacetic acid
LVI	Lymphovascular invasion
MMRd	Mismatch repair deficient
mRNA	Messenger ribonucleic acid
NA	Not available
NADPH	Nicotinamide adenine dinucleotide phosphate
NFκB	Nuclear Factor kappa-light-chain-enhancer of activated B cells
“none”	no resampling
NS	Non-significant
NSMP	No specific molecular profile
p53abn	p53 abnormal
PGF2 α	Prostaglandin F2α
POLEmut	POLE ultramutated
RA	Retinoic acid
REMARK	REporting recommendations for tumour MARKer prognostic studies
RNA	Ribonucleic acid
ROC	Receiver operating characteristic
ROSE	Random Over-Sampling Examples
ROUT	Robust regression and outlier detection
SD	Standard deviation
SMOTE	Synthetic Minority Over-Sampling Technique
SOP	Standard operating procedure
TCGA	The Cancer Genome Atlas
α (RARα)	Retinoic acid receptor α
