# Supplementary material for: AKR1B1 and AKR1B10 as Potential Prognostic Biomarkers of Endometrial Cancer
Source: Int J Mol Sci. 2026 Aug 26;27(17):7639. doi: 10.3390/ijms27177639 (PMC13566823; doi:10.3390/ijms27177639)
Supplement: Supplementary file 1 [file ijms-27-07639-s001.zip › ijms-4475370-supplementary.pdf]

AKR1B1 and AKR1B10 as Potential Prognostic Biomarkers of Endometrial Cancer

Maja Novak Pušić [1], Špela Smrkolj [2,3] and Tea Lanišnik Rižner [1,*]

Supplementary Materials

Supplementary Table S1. Reporting recommendations for tumour MARKer prognostic studies (REMARK) checklist

	Item to be reported	Page no.
**INTRODUCTION**		
1	State the marker examined, the study objectives, and any pre-specified hypotheses.	1, 2, 3
**MATERIALS AND METHODS**		
Patients		
2	Describe the characteristics (e.g., disease stage or co-morbidities) of the study patients, including their source and inclusion and exclusion criteria.	12
3	Describe treatments received and how chosen (e.g., randomized or rule-based).	NA
Specimen characteristics		
4	Describe type of biological material used (including control samples) and methods of preservation and storage.	13
Assay methods		
5	Specify the assay method used and provide (or reference) a detailed protocol, including specific reagents or kits used, quality control procedures, reproducibility assessments, quantitation methods, and scoring and reporting protocols. Specify whether and how assays were performed blinded to the study endpoint.	13
Study design		
6	State the method of case selection, including whether prospective or retrospective and whether stratification or matching (e.g., by stage of disease or age) was used. Specify the time period from which cases were taken, the end of the follow-up period, and the median follow-up time.	3, 4, 13, 14
7	Precisely define all clinical endpoints examined.	1
8	List all candidate variables initially examined or considered for inclusion in models.	13
9	Give rationale for sample size; if the study was designed to detect a specified effect size, give the target power and effect size.	13
Statistical analysis methods		
10	Specify all statistical methods, including details of any variable selection procedures and other model-building issues, how model assumptions were verified, and how missing data were handled.	13, 14
11	Clarify how marker values were handled in the analyses; if relevant, describe methods used for cutpoint determination.	6, 14
**RESULTS**		
Data		
12	Describe the flow of patients through the study, including the number of patients included in each stage of the analysis (a diagram may be helpful) and reasons for	3, 4

	dropout. Specifically, both overall and for each subgroup extensively examined report the numbers of patients and the number of events.	
13	Report distributions of basic demographic characteristics (at least age and sex), standard (disease-specific) prognostic variables, and tumor marker, including numbers of missing values.	4, 5 - Table 1.
Analysis and presentation		
14	Show the relation of the marker to standard prognostic variables.	5 - 10
15	Present univariable analyses showing the relation between the marker and outcome, with the estimated effect (e.g., hazard ratio and survival probability). Preferably provide similar analyses for all other variables being analyzed. For the effect of a tumor marker on a time-to-event outcome, a Kaplan-Meier plot is recommended.	8, 9, 10
16	For key multivariable analyses, report estimated effects (e.g., hazard ratio) with confidence intervals for the marker and, at least for the final model, all other variables in the model.	9 - Tables 2 and 3, Supp Material - Supp. Tables S3, S4
17	Among reported results, provide estimated effects with confidence intervals from an analysis in which the marker and standard prognostic variables are included, regardless of their statistical significance.	9, Table 2, 3
18	If done, report results of further investigations, such as checking assumptions, sensitivity analyses, and internal validation.	7, 8
**DISCUSSION**		
19	Interpret the results in the context of the pre-specified hypotheses and other relevant studies; include a discussion of limitations of the study.	10, 11, 12
20	Discuss implications for future research and clinical value.	10, 11, 12

Supplementary Table S2. The odds ratio for the logistic regression, with 5-fold cross-validation calculated to predict patients with Grade 1 – Grade 2 versus patients with Grade 3 endometrial cancer using AKR1B1, AKR1B10 or both AKR1B1 and AKR1B10

AKR1B10 and AKR1B1									
	**Odds_Ratio**	**2.5 %**	**97.5 %**	**z value**	**Pr(>|z|)**	**Estimate**	**Std. Error**	**lower CI - estimate**	**upper CI - estimate**
(Intercept)	0.0004	0.0000	0.1524	-2.3446	**0.0190**	-7.8735	3.3581	-14.4555	-1.2916
AKR1B1	1.0030	0.9972	1.0089	1.1517	0.2494	0.0030	0.0026	-0.0021	0.0082
AKR1B10	1.0034	1.0005	1.0074	1.9488	0.0513	0.0034	0.0017	0.0000	0.0067
AKR1B1: AKR1B10	1.0000	1.0000	1.0000	-1.3181	0.1875	0.0000	0.0000	0.0000	0.0000
**AKR1B1**									
	**Odds_Ratio**	**2.5 %**	**97.5 %**	**z value**	**Pr(>|z|)**	**Estimate**	**Std. Error**	**lower CI - estimate**	**upper CI - estimate**
(Intercept)	0.2793	0.0802	1.4981	-1.6843	0.0921	-1.2754	0.7572	-2.7596	0.2088
AKR1B1	0.9994	0.9976	1.0003	-0.7694	0.4417	-0.0006	0.0007	-0.0020	0.0009
**AKR1B10**									
	**Odds_Ratio**	**2.5 %**	**97.5 %**	**z value**	**Pr(>|z|)**	**Estimate**	**Std. Error**	**lower CI - estimate**	**upper CI - estimate**
(Intercept)	0.0065	0.0003	0.0596	-3.7534	**0.0002**	-5.0432	1.3436	-7.6767	-2.4097
AKR1B10	1.0016	1.0007	1.0029	2.8904	**0.0038**	0.0016	0.0006	0.0005	0.0027

Abbreviations: AKR1B1, aldo-keto reductase proteins family 1 member B1; AKR1B10, aldo-keto reductase proteins family 1 member B10; Pr(>|z|), two tailed p-value; CI, confidence interval. Bold values indicate statistically significant results (p < 0.05).

Supplementary Table S3. The odds ratio for the logistic regression, with 5-fold cross-validation calculated to predict patients with lymphovascular invasion versus patients without lymphovascular invasion using AKR1B1, AKR1B10 and clinical characteristics of patients

Feature	Odds ratio	2.5 %	97.5 %	z value	p-value	Estimate	Standard error	lower CI - estimate	upper CI - estimate
Age	9.881000e-01	0.8975	1.0870	-0.2505	0.8022	-0.0120	0.0479	-0.1060	0.0819
AKR1B1	1.000200e+00	0.9993	1.0014	0.4821	0.6297	0.0002	0.0005	-0.0007	0.0012
AKR1B10	9.992000e-01	0.9980	1.0001	-1.6044	0.1086	-0.0008	0.0005	-0.0018	0.0002
BMI	9.881000e-01	0.8897	1.0926	-0.2330	0.8158	-0.0120	0.0515	-0.1129	0.0889
Contraceptives Yes	4.060224e+09	0.0000	Inf	0.0013	0.9989	22.1245	16532.2341	-32381.0543	32425.3034
Hormonal_therapy Yes	0.000000e+00	NA	Inf	-0.0079	0.9937	-483.0625	61208.7507	-120452.2140	119486.0890
Parity	4.692000e-01	0.1784	1.0477	-1.7160	0.0862	-0.7566	0.4409	-1.6208	0.1076
Smoker - Past	3.163506e+208	0.0000	NA	0.0078	0.9937	480.0894	61208.7507	-119489.0620	120449.2408
Smoker -Yes	4.360000e-01	0.0312	5.0970	-0.6622	0.5079	-0.8300	1.2535	-3.2869	1.6269

Abbreviations: BMI, body mass index; AKR1B1, aldo-keto reductase proteins family 1 member B1; AKR1B10, aldo-keto reductase proteins family 1 member B10; CI, confidence interval.

Supplementary Table S4. Cox Proportional Hazards Model for recurrence analysis with lymphovascular invasion status as predictor and protein concentrations as groups

Variable	Hazard Ratio	Lower 95% CI	Upper 95% CI	p-value
Age	2.400935e+03	2.681487e-212	2.149735e+218	0.9754109
BMI	2.957430e+00	4.921961e-109	1.777014e+109	0.9932301
Smoker - Past	1.052292e-43	0.000000e+00	Inf	0.9972624
Smoker - Yes	4.286047e-01	0.000000e+00	Inf	0.9999966
Hormonal therapy - Yes	9.334745e+60	0.000000e+00	Inf	0.9444348
AKR1B10_groupless median	2.190401e-17	0.000000e+00	Inf	0.9860399
AKR1B1_groupless median	1.734283e-44	0.000000e+00	Inf	0.9583567
Parity	4.198246e+11	0.000000e+00	Inf	0.9783817
Lymphovascular invasion - Yes	2.007818e+10	0.000000e+00	Inf	0.9919698

Abbreviations: BMI, body mass index; AKR1B1, aldo-keto reductase proteins family 1 member B1; AKR1B10, aldo-keto reductase proteins family 1 member B10; CI, confidence interval. Inf, Infinity.

Supplementary Table S5. Cox Proportional Hazards Model for recurrence analysis without lymphovascular invasion status as predictor and protein concentrations as groups

Variable	Hazard Ratio	Lower 95% CI	Upper 95% CI	p-value
Age	5.840181e+10	4.032316e-215	8.458594e+235	0.9253325
BMI	1.030908e+03	1.807198e-102	5.880769e+107	0.9550418
Smoker - Past	4.268174e-138	0.000000e+00	Inf	0.9928605
Smoker - Yes	4.928208e+02	0.000000e+00	Inf	0.9999433
Hormonal therapy - Yes	3.628896e+203	0.000000e+00	Inf	0.8209754
AKR1B10_groupless median	7.009863e-29	0.000000e+00	Inf	0.9749535
AKR1B1_groupless median	2.538279e-130	0.000000e+00	Inf	0.8840612
Parity	9.493744e+37	0.000000e+00	Inf	0.9324693

Abbreviations: BMI, body mass index; AKR1B1, aldo-keto reductase proteins family 1 member B1; AKR1B10, aldo-keto reductase proteins family 1 member B10; CI, confidence interval. Inf, Infinity.

Platform-integrated analysis of publicly available data

To identify candidate genes and proteins for future multi-marker panel development, gene expression correlation analysis for AKR1B1 and AKR1B10 was performed using publicly available transcriptomic data from the TCGA PanCancer Atlas endometrial carcinoma dataset retrieved from cBioPortal (https://www.cbioportal.org/, accessed 15–17 November 2025, n=527). RNA-seq mRNA expression z-scores relative to all samples (log RNA Seq V2 RSEM) were used. Spearman correlation coefficients and associated p-values were extracted for all genes showing significant association with AKR1B1 or AKR1B10 expression. Results are presented in Supplementary Table S9.

Supplementary Table S6. Top 10 genes showing highest positive or negative correlation with *AKR1B1* and *AKR1B10* based on platform-based analysis of TCGA PanCancer mRNA expression data.

	Correlated Gene	Cytoband	Spearman's Correlation	p-Value	q-Value
AKR1B1	*UCHL1*	4p13	0.5734461118278691	2,11E-31	4,20E-28
	C9ORF152	9q31.3	-0.5167870215061791	2,54E-22	2,54E-18
	TMEM144	4q32.1	-0.5090151684593638	4,42E-22	2,94E-17
	CCNE1	19q12	0.5062817662997311	1,18E-19	5,91E-17
	RAE1	20q13.31	0.5042923244027482	2,42E-19	9,64E-17
	IL20RA	6q23.3	-0.4874472588304341	8,38E-19	2,79E-14
	MRGBP	20q13.33	0.4854070972857994	1,67E-17	4,22E-14
	CREB3L1	11p11.2	-0.4853588882672358	1,69E-16	4,22E-14
	SLC5A1	22q12.3	-0.4841358838167174	2,55E-16	5,38E-14
	SRSF12	6q15	0.48396705782655347	2,70E-16	5,38E-14
AKR1B10	*AKR1B15*	7q33	0.6620653596489857	9,09E-53	1,81E-47
	AKR1C2	10p15.1	0.4493009447709085	1,51E-11	1,51E-07
	AKR1C3	10p15.1	0.4345739793681693	1,09E-09	7,27E-08
	CYP4F3	19p13.12	0.4243887182951996	1,87E-09	9,33E-07
	NQO1	16q22.1	0.41969589101066307	6,70E-09	2,67E-04
	XDH	2p23.1	0.41882214801268197	8,47E-10	2,82E-05
	TMPRSS4	11q23.3	0.4055849494152126	2,76E-06	7,86E-04
	AKR1C1	10p15.1	0.40168803799561614	7,47E-07	1,86E-02
	PI3	20q13.12	0.39273545066438886	7,00E-06	1,55E-01
	CYP4F11	19p13.12	0.3919645096572393	8,47E-06	1,69E-02

Supplementary Figures

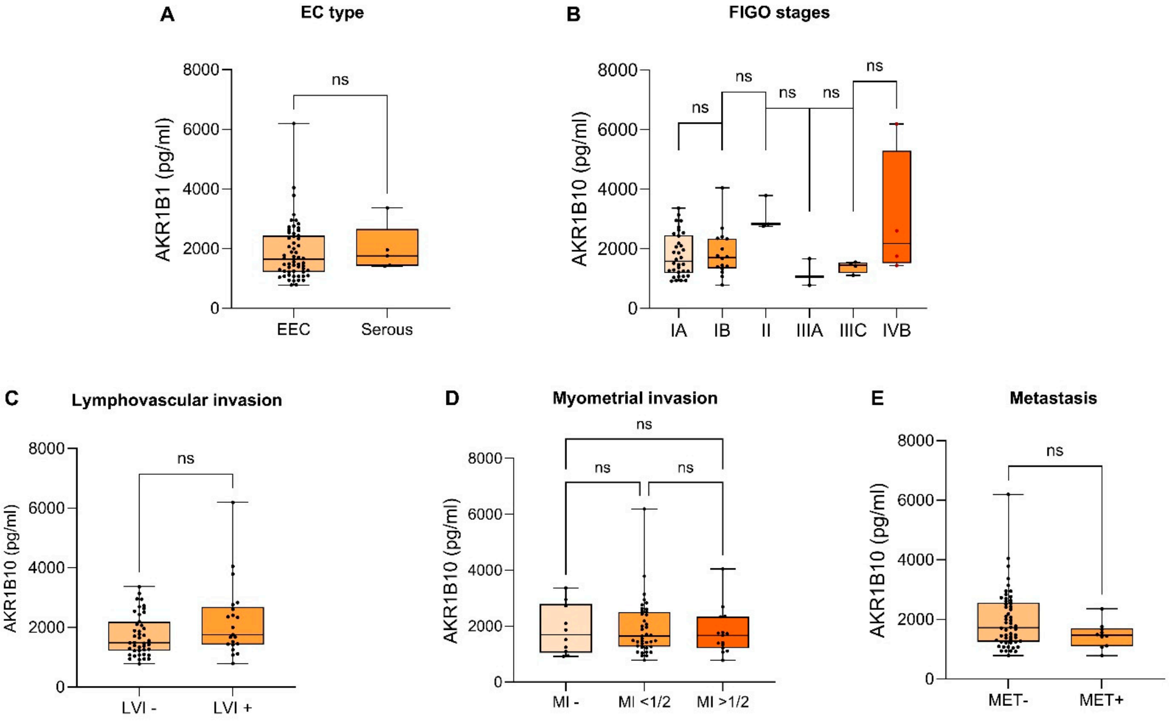

Supplementary Figure S1. Levels of AKR1B10 measured in plasma samples of endometrial cancer patients and analysed according to: A) endometrial cancer type, B) endometrial cancer stage, C) presence of lymphovascular invasion, D) depth of myometrial invasion and E) presence of metastases. Data are presented as box and whiskers plots with values from lower to upper quartiles (25th to 75th percentile) and median centred. Abbreviations: AKR1B1, aldo-keto reductase proteins family 1 member B1; AKR1B10, aldo-keto reductase proteins family 1 member B10; ns, non-significant; LVI – lymphovascular invasion; MET – metastasis; MI – myometrial invasion; FIGO, International Federation of Gynecology and Obstetrics; EC, endometrial cancer; EEC, endometrioid endometrial cancer

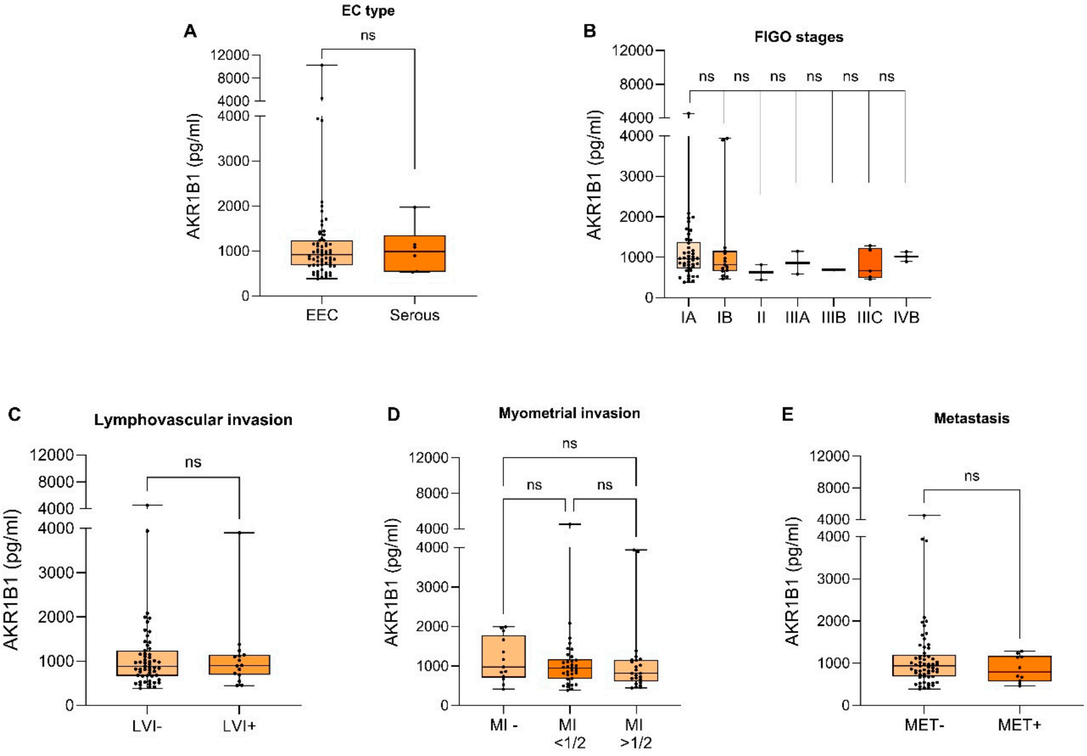

Supplementary Figure S2. Levels of AKR1B1 measured in plasma samples of endometrial cancer patients and analysed according to: A) endometrial cancer type, B) FIGO stage, C) presence of lymphovascular invasion, D) depth of myometrial invasion and E) presence of metastases. Data are presented as box and whiskers plots with values from lower to upper quartiles (25th to 75th percentile) and median centred. Abbreviations: AKR1B1, aldo-keto reductase proteins family 1 member B1; AKR1B10, aldo-keto reductase proteins family 1 member B10; ns, non-significant; LVI – lymphovascular invasion; MET – metastasis; MI – myometrial invasion; FIGO, International Federation of Gynecology and Obstetrics; EC, endometrial cancer; EEC, endometrioid endometrial cancer

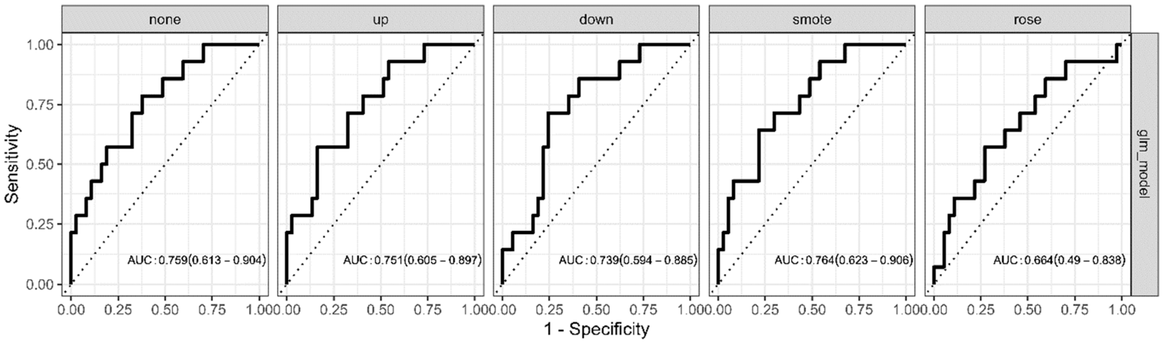

Supplementary Figure S3. Receiver Operating Characteristic (ROC) curves and Area Under the Curve (AUC) for the logistic regression model across different sampling methods used to predict lymphovascular invasion status in training set. Abbreviations: ROSE, random over-sampling examples; SMOTE, synthetic minority over-sampling technique; "none", no resampling; AUC, area under the curve; glm, generalized linear model
